# Association between Mood Disorders, Problematic Internet Use and Online Gambling Addiction: A Systematic Review

**DOI:** 10.1192/j.eurpsy.2022.2160

**Published:** 2022-09-01

**Authors:** E. Marimón Muñoz, E. Miranda Ruiz, A. Stoppa Montserrat

**Affiliations:** 1 CST, Psychiatry, Barcelona, Spain; 2 CST, Psychiatry, Terrassa, Spain

**Keywords:** bipolar disorder, problematic Internet use, internet gaming disorder, depressive disorder

## Abstract

**Introduction:**

New technologies have become widespread in the last decades, becoming an essential tool for today’s population. However, due to the increase in its use, multiple problems have surfaced at a psychopathological level.

**Objectives:**

The main goal of this study is to review, in an updated manner, the existing bibliography on the problematic use of the Internet and online gambling in the adult population and its relationship with Mood Disorders, exploring beyond Major Depressive Disorders so as to include Bipolar Disorders.

**Methods:**

A search was carried out in Medline, Tripdatabase and in the Virtual Health Library. We use the terms “Bipolar Disorder”, “Mood Disorders”; “Depressive disorders”; “Comorbidity”; “Problematic Internet use” and “Internet Gaming disorder”. Narrowing the search to the last 4 years and obtaining a total of 14 articles, of which only 10 were included after a thorough review.

**Results:**

A significant association was found between internet addiction in its different forms (Smartphone, Social Networks, Internet in general and IGD and MDD. A neuroanatomical correlation between Internet Gaming Disorder and Major Depressive Disorder was also established. A heterogeneity of criteria for addiction evaluation was observed. However, little information was found regarding the association between the addictive disorders and Bipolar Disorder.

**Conclusions:**

The correlation between the behavioral addiction forms previously mentioned and bipolar disorder must be further studied. There is a clear association between internet addiction and major depressive disorder. The established neuroanatomical correlation promotes the study of the applicability of brain stimulation techniques as a potential treatment for this type of pathology.

**Disclosure:**

No significant relationships.

